# Impact of Nonsurgical Periodontal Treatment of Periodontitis Stages 2 and 3 on Oral Health-Related Quality of Life

**DOI:** 10.3390/healthcare12141430

**Published:** 2024-07-17

**Authors:** Ali J. B. Al-Sharqi, Ali A. Abdulkareem, Sarhang Gul, Andrew Rawlinson

**Affiliations:** 1 Department of Periodontics, College of Dentistry, University of Baghdad, Bab Al Mudam, Baghdad P.O. Box 1417, Iraq; alijaafer@codental.uobaghdad.edu.iq; 2 Medical Laboratory Department, College of Health and Medical Technology, Sulaimani Polytechnic University, Sulaymaniyah P.O. Box 70-236, Iraq; sarhang.hama@univsul.edu.iq; 3 Department of Periodontics, College of Dentistry, University of Sulaimani, Sulaymaniyah 46001, Iraq; 4 Academic Unit of Restorative Dentistry, School of Clinical Dentistry, Claremont Crescent, Sheffield S10 2TA, UK; a.rawlinson@sheffield.ac.uk

**Keywords:** periodontitis, nonsurgical periodontal treatment, OHRQoL, clinical study

## Abstract

This study investigates the impact of nonsurgical periodontal treatment (NSPT) on oral health-related quality of life (OHRQoL) in patients with periodontitis stages (S)2 and S3, and the factors associated with the prediction of patient-reported outcomes. Periodontitis patients (n = 68) with moderately deep periodontal pockets were recruited. Responses to the Oral Health Impact Profile (OHIP)-14 questionnaire and clinical parameters including plaque index, bleeding on probing (BOP), probing pocket depth (PPD), and clinical attachment loss (CAL) were recorded. All patients received supra- and subgingival professional mechanical plaque removal. All clinical parameters and questionnaire responses were recorded again 3 months after NSPT. Clinical parameters and OHIP-14 scores for both stages of periodontitis were significantly improved 3 months after treatment. However, participants with periodontitis S3 had significantly higher total OHIP-14, physical pain, and functional limitation domains scores than periodontitis S2 cases. Baseline CAL, BOP, and the presence of PPD in anterior teeth were positively associated with increased OHIP-14 scores after NSPT. NSPT improved OHRQoL in participants with periodontitis S2 and S3. This was more pronounced in participants having periodontitis S3 than S2. Poorer OHRQoL could be anticipated in people having severe CAL, high BOP, and presence of pockets in the anterior teeth.

## 1. Introduction

Periodontitis is characterized by episodes of activity and remission, and is associated with progressive loss of tooth-supporting tissues [1]. Negative impacts of this disease can extend beyond the oral cavity to involve other distant organs, causing deterioration of not only the systemic health but psychological and mental health [2,3,4,5]. Periodontitis is one of the most common reasons for tooth loss, with devastating outcomes on oral function, aesthetics, and general wellbeing, together with adverse economic consequences [6,7].

Quality of life is a broad multidimensional term that has been described by the World Health Organisation (WHO) as “an individual’s perception of their position in life in the context of the culture and value systems in which they live and in relation to their goals, expectations, standards and concerns” [8]. Oral health-related quality of life (OHRQoL) is the part of quality of life which is affected by the healthy/diseased state of the oral cavity. Measurement of OHRQoL provides information reflecting the subjective perception of patients of their oral health and may be used to complement objective periodontal parameters measured by clinicians [9]. The majority of clinical studies measure clinical outcomes; however, the impact of patient-reported outcomes such as the need for re-treatment, tooth survival, and OHRQoL are seldom investigated in these studies [10]. OHRQoL is mainly measured by using self-reported questionnaires and the most commonly used one is the Oral Health Impact Profile (OHIP). The original or translated version of OHIP has been used in many countries [9,11].

Nonsurgical periodontal treatment (NSPT) is the gold-standard technique for treating shallow to moderately deep periodontal pockets with or without adjunctive [12,13,14,15,16]. Combining hyperbaric oxygen with NSPT for treating moderate to severe periodontitis has shown a significantly higher decrease in bleeding scores and a slower rate of bacterial recolonization than NSPT alone [17]. Similarly, clinical parameters exhibited marked improvement when NSPT was delivered in association with photodynamic therapy as compared to a control group [18]. The principle of NSPT is based on mechanically disrupting subgingival dysbiotic biofilm and decreasing the populations of pathobionts [19,20]. Application of topical agents such as sulfonated phenolics gel during NSPT enhanced favorable outcomes by mediating subgingival biofilm disaggregation [21]. The current evidence has highlighted the dilemma of assessing the outcomes of periodontal therapy. While the periodontist is seeking improvement in the clinical parameters, the patient may be looking for outcomes that are more meaningful to them, such as reducing tooth mobility, preserving the remaining teeth, comfortable mastication, and aesthetic outcomes [10]. Previous studies consistently report improvement of OHRQoL following periodontal treatment. OHIP scores significantly decreased following NSPT and the improvement was more pronounced in patients with deep periodontal pockets at baseline [9]. Results from other studies also showed that improvement in OHRQoL accompanied improvement in clinical parameters [22,23]. Results from a systematic review recommended using NSPT to improve both clinical and patient-based outcomes in the long term [12]. However, a recent comprehensive review has highlighted insufficiency of evidence related to the tangible outcomes after completion of active periodontal therapy [10]. Therefore, this study aimed to investigate the impact of NSPT on OHRQoL for patients with periodontitis stages (S)2 and S3, together with clinical and demographic factors associated with the prediction of perceived outcomes by those patients. 

## 2. Materials and Methods

### 2.1. Study Design and Eligibility Criteria

This prospective cohort was compiled with the World Medical Association Declaration of Helsinki and its later amendments. The ethical approval was obtained from the Ethics Committee, College of Dentistry, University of Baghdad (Ref. # 663, Date 13 September 2022). All study participants were verbally informed about the details of the study then asked to sign an informed consent before commencing any treatment. The participants were consecutively recruited from patients seeking periodontal treatment in the Teaching Dental Hospital, College of Dentistry, University of Baghdad. Three visits were assigned for each patient returned to recording baseline clinical parameters and delivering professional mechanical plaque removal (PMPR). The second visit was dedicated to full-mouth root surface debridement. The last visit was planned 3 months after termination of active periodontal therapy. The responses to the OHIP-14 questionnaire were recorded at the baseline visit and 3 months after NSPT.

The inclusion criteria were participants >18 years with no history of systemic disease such as diabetes mellitus and cardiovascular diseases, and non-smokers. The participants were educated to a level that enabled them to read and understand the OHIP-14 questionnaire. The included participants were diagnosed with periodontitis, defined by the presence of interproximal clinical loss of attachment (CAL) at ≥2 teeth or the presence of CAL ≥3 mm at the facial/oral surfaces associated with probing pocket depths (PPDs) ≥4 mm [24]. The severity of the periodontitis cases was further subclassified into S2 and S3: when interproximal CAL at the worst site was 3–4 mm; and when it was ≥5 mm, with radiographic bone loss extending to the coronal and mid-thirds or beyond, respectively [24]. Additionally, the cases were generalized as having >30% of teeth affected by CAL and with ≥10 occluding pairs of teeth. Only cases with moderately deep pockets (4–6 mm) scheduled for NSPT were included. 

### 2.2. Demographic Variables, Clinical Parameters, and Intervention

Demographic variables were recorded for each patient including the age, sex, income, occupation, educational achievement, and daily brushing frequency (twice daily). The threshold of low income (<5.5 USD per day) was determined according to a previously published report [25]. The educational achievement was dichotomized into those holding a college degree or equivalent and participants educated less than this level. The occupation was also recorded as a dichotomous variable as employed (part- or full-time paid job) or not employed (currently out of work or retired). 

This was followed by a full-mouth periodontal charting including plaque index (PI) [26], bleeding on probing (BOP) [27], PPD, CAL, and the number of missing teeth. In detail, a disclosing pellet (Biofilm Discloser, EMS, Nyon, Switzerland) was applied to the teeth and the plaque score was recorded. The periodontal probe (UNC-15, Medesy, Maniago, Italy) was inserted to the depth of the sulcus/pocket starting from the upper right molar and moving forward. Any sign of bleeding noticed after 20 s was recorded as 1 and absence of BOP was designated as 0. Simultaneously, PPD and CAL were measured, representing the linear distances from the gingival margin and cementoenamel junction, respectively, to the base of the sulcus/pocket. The cases were treated in accordance with treatment guidelines for periodontitis S2 and S3 issued by the European Federation of Periodontology [13]. At baseline, oral hygiene instructions were given together with supra- and subgingival PMPR using an ultrasonic scaler (Woodpecker, Ultrasonic Piezoelectric Scaler UDS-A, Guilin, China). After 1 week, participants underwent RSD using Gracey curettes (Medesy, Maniago, Italy) under local anesthesia. After completing treatment, patients were asked to return 3 months later for review and to repeat the measurement of clinical parameters. A site was considered unresponsive to treatment if it exhibited a PPD of 4 mm associated with BOP or a deep periodontal pocket (>6 mm) with or without bleeding [10]. All measurements were performed by the same calibrated examiner, using a UNC-15 periodontal probe (Medesy, Maniago, Italy). The level of consistency for categorical parameters (BOP and PI) was >80% using the kappa coefficient test and for continuous parameters (PPD and CAL) was >90%, as indicated by the interclass coefficient test. 

### 2.3. Questionnaire

The OHRQoL was assessed using an Arabic version of the OHIP-14 questionnaire which consisted of seven domains with two questions each, namely, functional limitation, physical pain, psychological discomfort, physical disability, psychological disability, social disability, and handicap [28]. The response to each question was based on a 5-point Likert scale (never, hardly ever, occasionally, often, very often) scored from 0 to 4, and the total score of OHIP-14 was calculated by the sum of all domains. Each patient was asked to fill in the questionnaire at the baseline visit before conducting any treatment, and 3 months after finishing active periodontal therapy. The original version of OHIP-14 was issued in English; therefore, it had to be translated to Arabic and tested for validity and reliability. First, the questionnaire was forward-translated by two independent translators, one of them was fully aware and the other was naïve about the concept and aim of the questionnaire. Discrepancies in translation at this step were discussed and resolved by the same translators. Then, this version was back-translated into the original language to resolve any misunderstandings or unclear wordings. A pilot study, including 30 participants, was conducted to ensure that the items retained the intended meaning of the original tool. The reliability of the final translated version of the questionnaire was determined using Cronbach’s alpha formula, which showed a consistency of 81%, indicating an adequate internal consistency. 

### 2.4. Outcomes and Sample Size

Pocket closure, PPD to ≤4 mm with no BOP, and improved OHRQoL at the endpoint of the study were specified as the primary outcomes. While reduction in BOP and PI, together with gain in CAL were designated as secondary outcomes. The a priori sample size was calculated using a chi-square analysis for pocket closure following periodontal therapy. The ratio of successful/unsuccessful was 1.6, which was estimated from a previous study [29]. The calculation was performed considering 0.05 as the α error probability and 80% power using the G*Power software for Windows (Universität Düsseldorf, Düsseldorf, Germany). The calculated sample size was 68 subjects, which was increased by 10% to 75 subjects to compensate for possible dropout for any reason. 

### 2.5. Statistical Analysis

Mean, standard deviation, frequency, and percent were used to descriptively express the data, which were then assessed for normality distribution using the Shapiro–Wilk test. Accordingly, paired and unpaired *t*-tests were used to compare intra- and intergroup changes in clinical parameters. While changes (endpoint–baseline) in the total scores of OHIP-14 and subdomains were tested by using the Wilcoxon signed-rank test and Mann–Whitney test for intergroup comparison between paired and unpaired sets of data, respectively. Comparisons of categorical variables were assessed by conducting a chi-square test. A multiple linear regression model was used to determine the correlation between OHRQoL scores (dependent variable) following NSPT, with independent demographic and clinical variables. Results of the inferential analyses were considered significant when *p* < 0.05. All statistical procedures were conducted by using SPSS (version 28.0, Chicago, IL, USA) and GraphPad Prism (version 9, Boston, MA, USA).

## 3. Results

**Figure 1:**
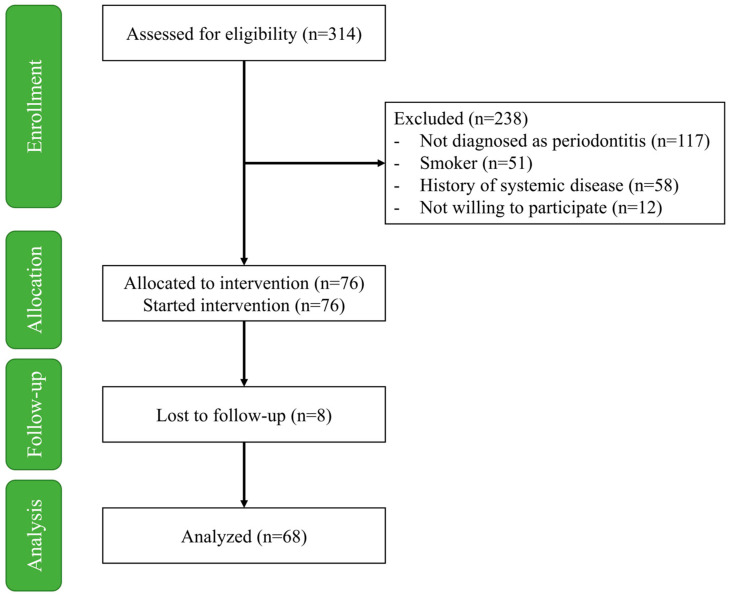
Flow diagram of the study.

The initial screening process involved 314 patients and the number of patients who completed the study was 68 (Figure 1). The demographic variables of the included patients are illustrated in Table 1. The final sample consisted of 36 (52.9%) males and 32 (47.1%) females, with an average age of 56.7 ± 7.2 years old. These patients were sub-classified according to stages of periodontitis and showed no significant difference according to age, gender, income, occupation, educational level, or daily brushing frequency. 

Comparisons of clinical periodontal parameters for all patients are illustrated in Table 2. All clinical parameters (PI, BOP, PPD, and CAL) and number of periodontal pockets were significantly improved 3 months after NSPT. 

A similar pattern was observed when comparing these parameters according to stages (S2 and S3), also showing significant improvements at the endpoint for both groups (Table 3). Intergroup comparisons, S2 vs. S3, showed no significant differences in PI and BOP; however, PPD, CAL, and number of periodontal pockets were significantly higher in the S3 group than their S2 counterparts at baseline. Although PI, BOP, and number of periodontal pockets demonstrated no significant changes between S2 and S3, the latter showed significantly higher improvement in reducing PPD and CAL gain than in S2. In fact, patients in the periodontitis S2 group showed further loss of attachment of about 0.2 mm at the endpoint. The number of missing teeth was not changed after treatment; therefore, no comparison was performed at the endpoint of the study (Table 3).

The total number of treated periodontal pockets (Table 4) was 1093, which were distributed across anterior region (n = 402, 36.8%), premolars (n = 364, 33.3%), and molar teeth (n = 327, 29.9%). The overall success rate was 74.0%, with anterior teeth exhibiting the lowest number of residual pockets (n = 55, 13.7%) followed by premolars (n = 94, 25.8%) and molars (n = 135, 41.3%). Further analysis according to stages showed more favorable response to NSPT in all tooth types and overall results in association with S2 periodontitis in comparison to their S3 counterparts (Table 4).

Analysis of responses to the OHIP-14 questionnaire indicated that for total OHIP-14 responses all domains significantly improved 3 months after NSPT. Similarly, comparisons of responses according to the stage of periodontitis showed that all domains of the OHIP-14 questionnaire were also significantly improved at the endpoint of the study as compared to baseline, except for social disability (Table 5). 

**Figure 2:**
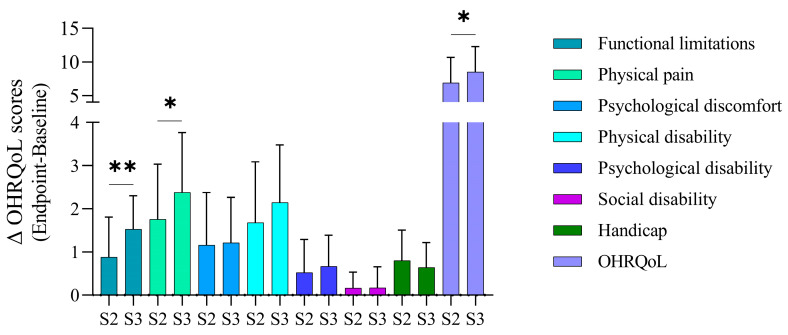
Intergroup comparison of OHIP-14 domains differences according to stage (S) of periodontitis 3 months after nonsurgical periodontal treatment (NSPT). Overall score showed significant improvement of S3 periodontitis after NSPT as compared to S2 patients. All domains were non-significantly different between the two groups except for functional limitations and physical pain domains, which were significantly higher in S3 than S2 periodontitis for the same period. Significant difference calculated by using Mann–Whitney test at * *p* < 0.05 and ** *p* < 0.002.

Intergroup comparison of changes in OHIP-14 scores (endpoint-baseline) between periodontitis groups S2 and S3 demonstrated significantly higher improvement in the total scores of periodontitis group S3 as compared to S2 3 months after NSPT. A sub-analysis indicated that both stages were significantly different only in association with functional limitations and physical pain components, while other domains showed no significant differences between the two stages (Figure 2).

A regression model showed that for every unit increase in BOP, CAL, and number of anterior teeth with PPD, the OHRQoL scores increased by 0.595, 0.288, and 0.186, respectively. Additionally, the findings suggest that both BOP, CAL, and number of teeth in the anterior segment with PPD can be used as predictors for the dependent variable, in which 58.1% of the variance in OHRQoL scores can be predicted from these parameters. In other words, the higher the severity of periodontitis at baseline, the higher the score of OHRQoL by the patient after NSPT (Table 6). 

## 4. Discussion

The current clinical study aimed to investigate the impact of NSPT on OHRQoL in periodontitis S2 and S3, which is known to adversely affect quality of life with moderately deep pockets. The results demonstrated significant improvement in total OHIP-14 scores for both stages at the endpoint of the study. Comparing both stages of periodontitis, this improvement was most noticeable in patients with a more severe form of periodontitis at the baseline, mainly in domains related to functional limitation and physical pain. These findings support the beneficial effect of NSPT not only on resolving periodontitis-associated inflammatory events but improving the quality of life for periodontitis patients. Additionally, proper periodontal therapy and strict maintenance programs lead to minimizing adverse systemic outcomes and increasing dental implant survival [30,31].

The success of NSPT was defined by a reduction in PPD to ≤4 mm and absence of BOP 3 months after completing active treatment [10]. The success rate of treating moderately deep pockets with NSPT was previously reported by a retrospective analysis [32]. The results indicated that NSPT resulted in success in one-third of cases and the highest rate of pocket closure was observed in the anterior teeth followed by premolars and molar teeth. The current study showed a similar pattern of successful outcomes at the endpoint, with a higher overall success rate of approximately 74%. This difference could be due to the case-definition of successful treatment, and the inclusion of smokers and severe periodontitis patients with deep periodontal pockets, who were excluded from the present study. Complementing objective clinical findings with the subjective experience of the participants by measuring OHRQoL is important when evaluating the success of periodontal therapy from both dentist and patient perspectives. To achieve this goal, OHIP-14 was developed and translated to different languages, and it showed constant sensitivity and reliability [33,34]. The same was observed in this study, in which OHIP-14 was translated to the native language of the targeted populations and showed a good level of reliability and consistency. 

Interestingly, in periodontitis S2 loss of attachment was observed 3 months after the completion of PMPR. According to the latest classification system of periodontal diseases, this stage of periodontitis is predominated by periodontal pockets ≤4 mm, mostly associated with horizontal bone loss [24]. Gunsolley et al. considered CAL in sites with minimal PPD after subgingival PMPR as a statistical phenomenon called regression towards the mean, which happens when a variable is too high or low and tends to move to the average upon the next measurement [35]. Another study, utilizing different types of curettes, concluded that manual root instrumentation inflicted an immediate average trauma of 0.76 mm relative loss of attachment regardless of instruments used [36].

A further debatable clinical finding was the plaque scores, which also significantly decreased after treatment. However, this did not prevent residual pocketing. Indeed, supra-gingival dental biofilm is more relevant to gingivitis and root caries, while subgingival biofilm is associated with periodontitis. The latter, once fully matured, is dominated by pathobionts independent of their growth requirement from supra-gingival biofilm and both compartments of dental biofilm are no longer considered as a continuum [37,38]. This could explain the ability of these pathogenic bacteria to re-populate the root surface after treatment even when supra-gingival biofilm control was adequate. 

The overall improved OHRQoL perceived by participants in the current study was consistent with a previous report [22]. Other studies also showed improvement in the pain domain [23,39] and functional limitation [40] following NSPT. These studies also indicated positive perceived outcomes in other domains such as psychological aspects, which were associated with a reduction in deep periodontal pockets. 

In this study, a linear logistic model indicated that high OHIP scores after treatment were positively correlated with BOP and CAL, together with the presence of periodontal pockets in the anterior teeth. This was evident from the significantly lower total OHIP-14 scores of periodontitis S2 compared to S3 at the endpoint of the study. Additionally, sub-analysis of the questionnaire domains demonstrated that functional limitations and physical pain score were significantly higher in periodontitis S3 than S2. These findings may be anticipated considering the amount of periodontal tissue loss is greater with increasing severity of disease, thus it is harder to repair or compensate for than in less severe periodontitis. Hence, the adverse effect on OHRQoL was more pronounced. The improvement in OHRQoL scores was more noticeable in participants with periodontitis S2 compared to S3, and this could be related to less periodontal tissue destruction in S2 at the baseline. Therefore, the changes in clinical parameters and OHRQoL scores were minimal and had perhaps less impact in S2 participants. Involvement of anterior teeth with periodontitis apparently had a greater impact on the outcomes reported by the patients. Anterior teeth have the greatest impact on the aesthetic and psychological values, and these domains may be compromised by the presence of increased gingival embrasures, increased tooth mobility, and drifting, which are a common consequence of progressive periodontal diseases [41]. A receding interdental papilla may also be responsible for phonetic problems and food impaction, which may have adverse social and psychological effects. Indeed, a previous study involving digitally manipulated images showing different esthetic problems demonstrated that black triangles were the third most disliked problem after dental caries and defective crown margins [42]. Additionally, proper function of masticatory apparatus starts with sound, periodontally healthy anterior teeth [43]. Therefore, loss of periodontal support may be responsible for uncomfortable chewing and pathologic drifting of these teeth [44], which is potentially responsible for limitations in masticatory function. 

Periodontal treatment is not only centered around improving clinical parameters but enhancing the OHRQoL domains. Unfortunately, the available literature provides sparse information about tangible patient-reported outcomes after periodontal treatment. Therefore, assessing the success of active periodontal treatment should be multidimensional by linking clinical outcomes with patient-reported perceived outcomes. Profiling subgingival microbiota, inclusion of more severe periodontitis cases, controlling glycemic state, and smoking cessation are other aspects that should be further investigated in future studies, and with a longer follow-up period. However, the current study provides insight about the impact of NSPT on people with periodontitis S2 and S3 by the inclusion of an OHRQoL measure. In addition, predictors influencing the tangible patient outcomes were also highlighted. 

Whilst the present study provides useful insight into the impact of NSPT on OHRQoL, caution should be exercised in the generalization of these findings. Future studies should be undertaken in other settings to further elucidate the impact of NSPT on tangible patient-reported outcomes. 

## 5. Conclusions

NSPT improved OHRQoL in participants with periodontitis stages 2 and 3. This was more pronounced in participants having S3 than S2 periodontitis. Poorer OHRQoL outcomes could be anticipated in people having severe CAL, high BOP, and presence of pockets in the anterior teeth. These results provide further support that earlier treatment of periodontitis is more favorably associated with improved OHRQoL.

## Figures and Tables

**Table 1:** Demographic variables of the study population for periodontitis stages 2 and 3.

Variables	Total	Stage 2	Stage 3	*p* Value
**Age (years) ^†^**	56.7 ± 7.2	58.0 ± 7.3	56.1 ± 7.3	0.33 *
**Sex ^§^**				
**Male**	32, 47.1%	14, 56.0%	21, 48.8%	0.62 **
**Female**	36, 52.9%	11, 44.0%	22, 51.2%	
**Income ^§^**				
**Low (<5.5 USD/day)**	25, 36.8%	8, 32.0%	17, 39.5%	0.60 **
**Middle (>5.5 USD/day)**	43, 63.2%	17, 68.0%	26, 60.5%	
**Occupation ^§^**				
**Employed**	33, 48.5%	19, 76.0%	24, 55.8%	0.12 **
**Not employed**	35, 51.55	6, 24.0%	29, 44.2%	
**Education ^§^**				
**<College level**	27, 39.7%	8, 32.0%	20, 46.5%	0.32 **
**College level or equivalent**	41, 60.3%	17, 68.0%	23, 55.5%	
**Daily brushing frequency (2x/day) ^§^**				
**Yes**	42, 61.8%	17, 68.0%	25, 58.1%	0.45 **
**No**	26, 38.2%	8, 32.0%	18, 41.9%	
**Total ^§^**	68, 100.0%	25, 100.0%	43, 100.0%	

^†^ Mean ±SD; ^§^ frequency, percent; * significant difference at *p* < 0.05 by *t*-test between periodontitis stages 2 and 3; ** significant difference at *p* < 0.05 by chi-square test between periodontitis stages 2 and 3.

**Table 2:** Comparisons of clinical parameters (mean ± standard deviation) at baseline and endpoint of the study.

	Baseline	Endpoint ^‡^	*p* Value *
**Periodontitis**			
**PI**	48.8 ± 7.8	13.4 ± 5.1	<0.001
**BOP**	52.5 ± 8.4	11.2 ± 4.4	<0.001
**PPD**	2.8 ± 0.3	2.5 ± 0.2	<0.001
**CAL**	2.7 ± 1.3	3.0 ± 2.5	<0.001
**Number of pockets/patient**	16.3 ± 2.8	4.2 ± 1.9	<0.001

PI: plaque index, BOP: bleeding on probing, PPD: probing pocket depth, CAL: clinical attachment loss. ^‡^ Three months after nonsurgical periodontal therapy. * Significant difference at *p* < 0.05 using paired *t*-test.

**Table 3:** Comparison of clinical parameters (mean ± standard deviation) according to stage of periodontitis at baseline and endpoint of the study.

		Baseline	Endpoint ^‡^	*p* Value *
**PI**	S2	47.5 ± 8.3	13.5 ± 6.2	<0.001
	S3	49.3 ± 7.6	13.2 ± 4.5	<0.001
*p* value **		0.38	0.45	
**BOP**	S2	51.3 ± 9.9	14.4 ± 5.4	<0.001
	S3	52.8 ± 7.5	12.6 ± 5.1	<0.001
*p* value **		0.49	0.23	
**PPD**	S2	2.6 ± 0.2	2.4 ± 0.3	0.009
	S3	2.9 ± 0.3	2.5 ± 0.2	<0.001
*p* value **		<0.001	0.03	
**CAL**	S2	2.5 ± 0.1	2.7 ± 0.3	0.05
	S3	2.8 ± 0.3	2.6 ± 0.1	0.002
*p* value **		<0.001	0.001	
**Number of pockets**	S2	14.9 ± 2.5	3.8 ± 2.1	<0.001
	S3	17.4 ± 2.8	4.6 ± 1.7	<0.001
*p* value **		<0.001	0.07	
**Missing teeth**	S2	3.2 ± 0.6	-	
	S3	5.2 ± 1.1	-	
*p* value **		<0.001		

PI: plaque index, BOP: bleeding on probing, PPD: probing pocket depth, CAL: clinical attachment loss, S: stage, ^‡^ Three months after nonsurgical periodontal therapy. Significant difference at *p* < 0.05 using * paired *t*-test for endpoint versus baseline and ** unpaired *t*-test for S2 vs. S3.

**Table 4:** Distribution of residual pockets and success rate of nonsurgical periodontal therapy according to tooth type at the endpoint of the study.

		Anterior Teeth	Premolars	Molars	Total
**Periodontitis ^†^**	Residual pockets	55, 13.7%	94, 25.8%	135, 41.3%	284, 26.0%
	Success rate	347, 86.3%	270, 74.2%	192, 58.7%	809, 74.0%
**S2 ^†^**	Residual pockets	19, 13.0%	33, 23.7%	57, 38.8%	109, 25.2%
	Success rate	127, 87.0%	106, 76.3%	90, 61.2%	323, 74.8%
**S3 ^†^**	Residual pockets	36, 14.1%	61, 27.1%	78, 43.3%	175, 26.5%
	Success rate	220, 85.9%	164, 72.9%	102, 56.7%	486, 73.5%
	Total	402, 36.8%	364, 33.3%	327, 29.9%	1093, 100%
**FI**	Residual pockets	-	-	62, 60.8%	-
	Success rate	-	-	40, 39.2%	-
	Subtotal	-	-	102, 31.2%	-

S: Stage; FI: furcation involvement; ^†^ frequency, percent.

**Table 5:** Intragroup comparisons of OHIP-14 domain scores between baseline and endpoint of the study.

		Total OHIP-14	D1	D2	D3	D4	D5	D6	D7
**Periodontitis ^†^**	Baseline	26.7 ± 5.4	4.2 ± 1.2	5.4 ± 1.4	3.5 ± 1.3	5.0 ± 1.2	3.2 ± 0.9	2.2 ± 0.4	3.1 ± 0.8
	Endpoint	18.8 ± 1.9	3.0 ± 0.7	1.7 ± 0.4	2.3 ± 0.5	3.1 ± 0.7	2.6 ± 0.6	2.1 ± 0.2	2.5 ± 0.5
	*p* value *	<0.001	<0.001	<0.001	<0.001	<0.001	<0.001	0.03	<0.001
**Stage 2 ^†^**	Baseline	25.1 ± 5.2	3.8 ± 1.2	4.7 ± 1.3	3.4 ± 1.4	4.5 ± 1.2	3.2 ± 0.9	2.2 ± 0.5	3.1 ± 0.7
	Endpoint	18.2 ± 1.8	2.8 ± 0.8	3.0 ± 0.7	2.3 ± 0.4	2.8 ± 0.6	2.6 ± 0.5	2.0 ± 0.2	2.4 ± 0.5
	*p* value *	<0.001	<0.001	<0.001	<0.001	<0.001	0.006	0.12	<0.001
**Stage 3 ^†^**	Baseline	27.6 ± 5.4	4.5 ± 1.1	5.7 ± 1.4	3.5 ± 1.2	5.3 ± 1.1	3.2 ± 0.9	2.1 ± 0.4	3.1 ± 0.8
	Endpoint	19.1 ± 1.9	3.0 ± 0.7	3.3 ± 0.5	2.4 ± 0.4	3.2 ± 0.8	2.6 ± 0.6	2.0 ± 0.2	2.5 ± 0.5
	*p* value *	<0.001	<0.001	<0.001	<0.001	<0.001	<0.001	0.23	<0.001

OHRQoL: oral health-related quality of life, D1: functional limitations, D2: physical pain, D3: psychological discomfort, D4: physical disability, D5: psychological disability, D6: social disability, D7: handicap. ^†^ Mean ± SD, * significant difference at *p* < 0.05 by using Wilcoxon signed-rank test.

**Table 6:** Stepwise multiple linear regression model for clinical parameters as predictor of oral health-related quality of life outcome.

Variable ^†^	B ^‡^	SE ^‡^	*p* Value *
**Education**	0.411	0.249	0.10
**Income**	0.360	0.308	0.24
**Job**	0.318	0.487	0.52
**PI**	0.003	0.024	0.91
**PPD**	0.343	0.788	0.67
**Age**	−0.023	0.018	0.20
**Missing teeth**	0.192	0.123	0.12
**BOP**	0.595	0.016	>0.001
**CAL**	0.288	0.419	0.001
**Anterior teeth**	0.186	0.038	0.02
**Premolars**	0.005	0.084	0.84
**Molars**	−0.087	0.083	0.41
**Model summary**			
**R**	0.775		
**R^2^**	0.581		
**Adjusted R^2^**	0.552		

PI: plaque index, BOP: bleeding on probing, PPD: probing pocket depth, CAL: clinical attachment loss, SE: standard error. ^†^ Dependent variable: OHRQoL; ^‡^ unstandardized coefficients; * significant difference at *p* < 0.05. Significant differences indicated by a bold font.

## Data Availability

The raw data supporting the conclusions of this article will be made available by the authors on request.
